# Histone deacetylase inhibitor, suberoylanilide hydroxamic acid (SAHA), enhances anti-tumor effects of the poly (ADP-ribose) polymerase (PARP) inhibitor olaparib in triple-negative breast cancer cells

**DOI:** 10.1186/s13058-015-0534-y

**Published:** 2015-03-07

**Authors:** Ahrum Min, Seock-Ah Im, Debora Keunyoung Kim, Sang-Hyun Song, Hee-Jun Kim, Kyung-Hun Lee, Tae-Yong Kim, Sae-Won Han, Do-Youn Oh, Tae-You Kim, Mark J O’Connor, Yung-Jue Bang

**Affiliations:** 1 Cancer Research Institute, Seoul National University College of Medicine, Seoul, 110-799 Korea; 2 Department of Internal Medicine, Seoul National University College of Medicine, Seoul, 110-799 Korea; 3 Biomedical Research Institute, Seoul National University Hospital, Seoul, 110-799 Korea; 4 Seoul International School, Seongnam, 461-830 Korea; 5 Department of Internal Medicine, Chung Ang University College of Medicine, Seoul, 156-755 Korea; 6 AstraZeneca UK Ltd, Macclesfield, Cheshire, SK10 2NA UK

## Abstract

**Introduction:**

Olaparib, a poly (ADP-ribose) polymerase (PARP) inhibitor, has been found to have therapeutic potential for treating cancers associated with impaired DNA repair capabilities, particularly those with deficiencies in the homologous recombination repair (HRR) pathway. Histone deacetylases (HDACs) are important for enabling functional HRR of DNA by regulating the expression of HRR-related genes and promoting the accurate assembly of HRR-directed sub-nuclear foci. Thus, HDAC inhibitors have recently emerged as a therapeutic agent for treating cancer by inhibiting DNA repair. Based on this, HDAC inhibition could be predicted to enhance the anti-tumor effect of PARP inhibitors in cancer cells by blocking the HRR pathway.

**Methods:**

We determined whether suberoylanilide hydroxamic acid (SAHA), a HDAC inhibitor, could enhance the anti-tumor effects of olaparib on breast cancer cell lines using a cytotoxic assay, cell cycle analysis, and Western blotting. We evaluated how exposure to SAHA affects the expression of HRR-associated genes. The accumulation of DNA double strand breaks (DSBs) induced by combination treatment was assessed. Induction of autophagy was monitored by imaging green fluorescent protein-tagged microtubule-associated protein 1A/1B-light chain 3 (LC3) expression following co-treatment with olaparib and SAHA. These *in vitro* data were validated *in vivo* using a human breast cancer xenograft model.

**Results:**

Triple-negative breast cancer cell (TNBC) lines showed heterogeneous responses to the PARP and HDAC inhibitors. Co-administration of olaparib and SAHA synergistically inhibited the growth of TNBC cells that expressed functional Phosphatase and tensin homolog (PTEN). This effect was associated with down-regulation of the proliferative signaling pathway, increased apoptotic and autophagic cell death, and accumulation of DNA damage. The combined anti-tumor effect of olaparib and SAHA was also observed in a xenograft model. These data suggest that PTEN expression in TNBC cells can sensitize the cell response to simultaneous inhibition of PARP and HDAC both *in vitro* and *in vivo*.

**Conclusion:**

Our findings suggest that expression of functional PTEN may serve as a biomarker for selecting TNBC patients that would favorably respond to a combination of olaparib with SAHA. This provides a strong rationale for treating TNBC patients with PTEN expression with a combination therapy consisting of olaparib and SAHA.

**Electronic supplementary material:**

The online version of this article (doi:10.1186/s13058-015-0534-y) contains supplementary material, which is available to authorized users.

## Introduction

Breast cancer is a disease with a number of diverse morphological subtypes. Invasive ductal carcinoma is the most common morphologic subtype representing 80% of invasive breast cancer cases [1]. In addition, it can be subclassified into three major categories according to different expression levels of estrogen receptor (ER), progesterone receptor (PR), and human epidermal growth factor receptor-2 (HER2) [2]. In general, hormone receptor-positive breast cancer subtypes are less progressive and amenable to hormone therapy. Although HER2+ breast cancer subtype shows rapid progression, targeted therapy for treating breast cancers over-expressing HER2 has improved survival for HER2+ breast cancer patients [3]. In contrast to these two subtypes, triple-negative breast cancer (TNBC) is resistant to various chemotherapy agents and targeted drugs because a widely available target for this subtype has not yet been discovered. Therefore, the development of new combined targeted therapy and identification of biomarkers that can help predict responses to treatment are still major challenges in TNBC.

Recent progress in the field of DNA repair has demonstrated that a synthetic lethal approach involving the use of poly (ADP-ribose) polymerase (PARP) inhibitors is a promising new therapeutic strategy for treating various cancers. DNA repair inhibitors have been shown to work as single agents in patients with DNA repair-defective tumors [4,5]. The most notable example so far is the use of PARP inhibitors to treat individuals with inherited breast and ovarian cancers lacking wild-type copies of the *BRCA1* and *BRCA2* genes [6-8]. PARP inhibitors have also produced promising results in TNBC patients harboring *BRCA*-like genotypes or so-called BRCAness [9]. Therefore, development of strategies for using PARP inhibitors and selecting populations within TNBC that will respond favorably to PARP inhibitor treatment based on predictive biomarkers represents both a challenge and an opportunity for breast cancer research. Additionally, enzyme-mediated DNA repair can cause resistance to DNA-damaging anticancer drugs and radiation, and inhibition of DNA repair may be therapeutically beneficial. In particular, it has been observed that combining chemotherapy or radiotherapy with PARP inhibitors kills human cancer cells more effectively than a genotoxic agent alone [8,10]. The development of new therapies including molecular targeting agents is eagerly awaited as well as treatment strategies to overcome chemoresistance using PARP inhibitors that are effective for ameliorating TNBC.

During the past few years, histone deacetylases (HDACs) have garnered great interest as anticancer therapeutic targets. Experimental data have suggested that HDACs are involved in mammary tumorigenesis at multiple levels [11,12]. HDACs participate in the negative regulation of genes such as ones encoding cell cycle inhibitors, differentiation factors, and pro-apoptotic factors. In addition, the expression of genes associated with angiogenesis along with cell invasion and migration are enhanced by HDACs. Thus, HDACs play important roles in cancer development by regulating the expression of numerous genes involved in both cancer initiation and progression. Based on the role of HDACs in cancer development, HDAC inhibition could have potent anti-tumor effects on various types of cancer by affecting tumor cells at multiple levels. More specifically, inhibition by HDACs could induce cell cycle arrest, apoptosis, and differentiation while inhibiting angiogenesis along with cell migration and invasion [12].

HDACs also enable functional HRR by regulating the expression of homologous recombination repair (HRR)-related genes and promoting the accurate assembly of HRR-directed sub-nuclear foci [13,14]. There is evidence showing that dysfunctional HDACs lead to the downregulated expression of DNA repair genes including *RAD51* and *BRCA1/2*, resulting in defective DNA repair which can result in the accumulation of DNA damage [14,15]. HDAC inhibitors have thus emerged recently as a class of anticancer therapeutic agents that prevent DNA repair. HDAC inhibition sustains DNA damage signaling and suppresses DNA repair gene expression, which can increase the sensitivity of cells to DNA damaging agents similar to BRCA deficiency in breast cancer. For this reason, HDAC inhibition could enhance the anti-tumor effect of PARP inhibitors in cases of TNBC by blocking the DNA repair pathway. Previous studies have shown that HDAC inhibition does enhance cellular sensitivity to DNA damaging agents; however, specific markers that can help predict the combinational effect have not yet been identified [16-18].

In the present investigation, we identified a determinant of the combined effects of a PARP inhibitor with an HDAC inhibitor in TNBC cell lines. We evaluated one possible combined strategy to treat the TNBC subtype. We discovered that suberoylanilide hydroxamic acid (SAHA), a pan-HDAC inhibitor, enhanced the growth inhibitory activities of olaparib, a PARP inhibitor, in TNBC cells. Additionally, the combination of olaparib plus SAHA induced the accumulation of DNA DSBs and downregulated signal transduction in TNBC cells that expressed phosphatase and tensin homolog (PTEN). Our results suggest that the expression of PTEN in TNBC cells significantly increased the anti-tumor effects of olaparib and SAHA through the induction of both apoptotic and autophagic cell death. Using a xenograft mouse model of TNBC cells expressing PTEN, we verified that co-treatment with olaparib and SAHA inhibited tumor growth. Taken together, these data suggest that the combination of olaparib with SAHA exerts a synergistic effect on TNBC cells that is associated with increased levels of both apoptosis and autophagy regulated by PTEN. More importantly, our results provide a rationale for conducting future clinical trials evaluating the effectiveness of using olaparib combined with SAHA to treat TNBC patients.

## Methods

### Reagents

Olaparib was provided by AstraZeneca (Macclesfield, UK) and SAHA was purchased from Selleck (Houston, TX, USA). Both reagents were dissolved in dimethyl sulfoxide (DMSO) as 10 mmol/L stock solutions.

### Cell lines and culturing

Human breast cancer cells (MDA-MB-157, -231, -453, -468, BT-549, MCF7, T47D, SK-BR-3, HCC70, HCC1143, and Hs578T) whose identity was authenticated with a short tandem repeat analysis were purchased from the American Type Culture Collection (ATCC; Manassas, VA, USA). All cell lines were banked and passaged for less than 6 months before use, and were maintained in a humidified atmosphere containing 5% CO_2_ at 37°C in RPMI-1640 (Thermo Fisher Scientific Inc., Waltham, MA, USA) supplemented with 10% FBS; Welgene, Inc., Daegue, South Korea) and 10 μg/mL gentamicin (Cellgro, Manassas, VA, USA).

### Cell growth inhibition assay

An MTT assay was used to determine cell viability as previously described [19]. Cells were seeded at a density of 3 to 8 × 10^3^ cells per well in 96-well plates and incubated overnight at 37°C. The cells were then treated with either olaparib or SAHA alone or with a combination of olaparib and SAHA at specific concentrations for 5 d. After treatment with the drugs, MTT solution was added to each well and the plates were incubated for 4 h at 37°C before the medium was removed. After dissolving the resulting formazan crystals with DMSO, cell viability was evaluated by measuring the absorbance of each well at 540 nm with a VersaMax™ microplate reader (Molecular Devices, Sunnyvale, CA, USA). The combined effect of olaparib and SAHA was assessed using Calcusyn software (Biosoft, Cambridge, UK). The combination index (CI), which is used to evaluate the effect of two-drug combinations, was calculated using the Chou-Talalay method [20]. Drug synergism is defined by CI values <1 while antagonism is indicated by values >1.

### Western blot analysis

Protein expression levels were measured by western blotting as previously described [20]. Primary antibodies against MRE11, caspase3, PTEN, AKT, phosphorylated (p)-AKT, ERK, p-ERK, STAT3, p-STAT3, and LC3B were acquired from Cell Signaling Technology (Beverley, MA, USA). Anti-RAD51C (2H11/6) antibody was purchased from Novus Biologicals (Littleton, CO, USA). Antibodies against p21 and Beclin-1 were obtained from Abcam (Cambridge, UK). Anti-p-histone H2A.X antibody (clone JBW301) was acquired from Millipore (Billerica, MA, USA) while anti-PARP antibody was purchased from BD Biosciences (Bedford, MA, USA). Anti-α-tubulin antibody (Sigma Aldrich, St Louis, MO, USA) was used as a control.

### Cell cycle analysis

Cells treated with olaparib and/or SAHA were harvested, fixed in 70% ethanol, and then stored at -20°C. The cells were dissolved in 10 μg/mL RNase A (Sigma Aldrich) at 37°C for 20 minutes. Next, the cells were treated with 20 μg/mL propidium iodide (Sigma Aldrich) and the DNA contents of the cells (10,000 cells per experimental group) were measured using a fluorescence-activated cell sorting (FACS) Calibur flow cytometer (BD Biosciences).

### Plasmid and siRNA transfection

The pcDNA3.1-PTEN expression plasmid was obtained from the Korea Human Gene Bank (Seoul, South Korea) and the GFP-LC3 construct was purchased from Cell Biolabs (San Diego, CA, USA). siRNA specific for PTEN and nonspecific controls were purchased from Qiagen (Hilden, Germany). Transfection was conducted using Lipofectamine 2000 (Invitrogen, Carlsbad, CA, USA) according to the manufacturer’s instructions. The sequence of the PTEN-specific siRNA was 5′-AAGGCGTATACAGGAACAATA-3′. The sequence of the control (nonspecific) siRNA was 5′-AATTCTCCGAACGTGTCACG-3′.

### Comet assays

An alkaline comet assay using a Trevigen Comet assay kit (Trevigen, Gaithersburg, MD, USA) was performed following the manufacturer’s protocol. Tail lengths were measured with the Comet assay IV program (Andor technology, Belfast, UK).

### Immunofluorescence assay (GFP-LC3 localization)

Cells were plated on coverslips and transfected with the GFP-LC3 construct. After 2 d, the cells were fixed in 3.7% paraformaldehyde and permeabilized with 0.5% Triton X-100 in PBS (PBS-T). The coverslips were mounted onto slides using Faramount aqueous mounting medium (Dako, Glostrup, Denmark). Immunofluorescence was visualized using a Zeiss LSM 510 laser scanning microscope.

### Immunohistochemistry and terminal deoxynucleotidyl transferase dUTP nick end labeling (TUNEL) assay

Immunohistochemistry and a TUNEL assay were performed as previously described [19].

### *In vivo* study

All animal experiments were carried out in the animal facility of Seoul National University (Seoul, South Korea) in accordance with institutional guidelines and prior approval from the Institutional Animal Care and Use Committee (IACUC) committee. To measure the *in vivo* activity of olaparib and/or SAHA, 35 female Balb/c athymic nude 5-wk-old mice were purchased from Central Lab Animal Inc. (Seoul, South Korea). MDA-MB-231 cells (1 × 10^8^) were subcutaneously injected into each mouse. After implantation of the tumor cells, the size of the resulting tumors and body weight of each mouse were measured. When the tumor volume reached 200 mm^3^, the mice were randomly divided into different treatment groups (eight mice per group) and received vehicle, olaparib, SAHA, or a combination of olaparib and SAHA. All drugs were administered via oral gavages once daily at a concentration of 30 mg/kg for 28 consecutive days. Tumor volume was calculated using the following formula:

$$ \left({\left(\mathrm{width}\right)}^2\times \left(\mathrm{height}\right)\right)/2. $$

At the end of the measurement period, the mice were sacrificed with CO_2_ and the tumors were excised for further analysis.

### Statistical analysis

Data were analyzed using SigmaPlot version 9.0 (Systat Software Inc., San Jose, CA, USA). All results are expressed as the mean ± standard error (SE). The two-sided Student’s *t*-test was used when appropriate. *P*-values <0.05 were considered statistically significant.

## Results

### Breast cancer cells have different levels of sensitivity to olaparib or SAHA alone

**Figure 1:**
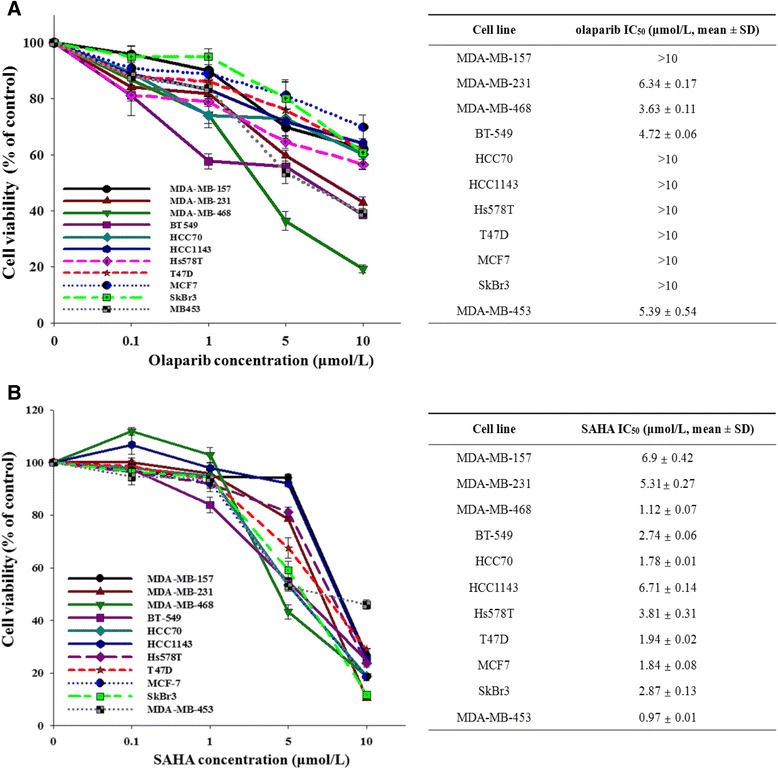
**Breast cancer cell lines show a heterogeneous response to olaparib and SAHA.** The growth inhibitory effects of olaparib and suberoylanilide hydroxamic acid (SAHA) were measured with an MTT assay. The cells were treated with increasing doses of olaparib **(A)** and SAHA **(B)** for 5 d. The percentage of surviving cells is presented in a graph with SD bars (n = 3; right). IC_50_ values were calculated using SigmaPlot and are shown in the table (left).

To assess the anti-proliferative effects of olaparib and SAHA on human breast cancer cells, 11 human breast cancer cell lines were exposed to different concentrations of olaparib or SAHA. Cell proliferation and biological activity were analyzed using an MTT assay (Figure 1A and B). Results of this assay indicate that breast cancer cells have a heterogeneous response to olaparib and SAHA regardless of subtype.

Recent studies have suggested that HDAC inhibition leads to the downregulated expression of DNA repair genes [14,15,18,21,22]. We therefore evaluated the effects of HDAC inhibition by SAHA in the regulation of protein expressions that are known to effect the sensitivity to PARP inhibitors, such as DNA repair factors. SAHA markedly increased the levels of PTEN and p21. In contrast, the expression of MRE11 and RAD51 was downregulated (Additional file 1: Figure S1). These findings imply that SAHA can affect the DNA damage response by suppressing HRR gene expression.

### Co-administration of SAHA and olaparib has a synergistic anti-proliferative effect on some TNBC cell lines

**Figure 2:**
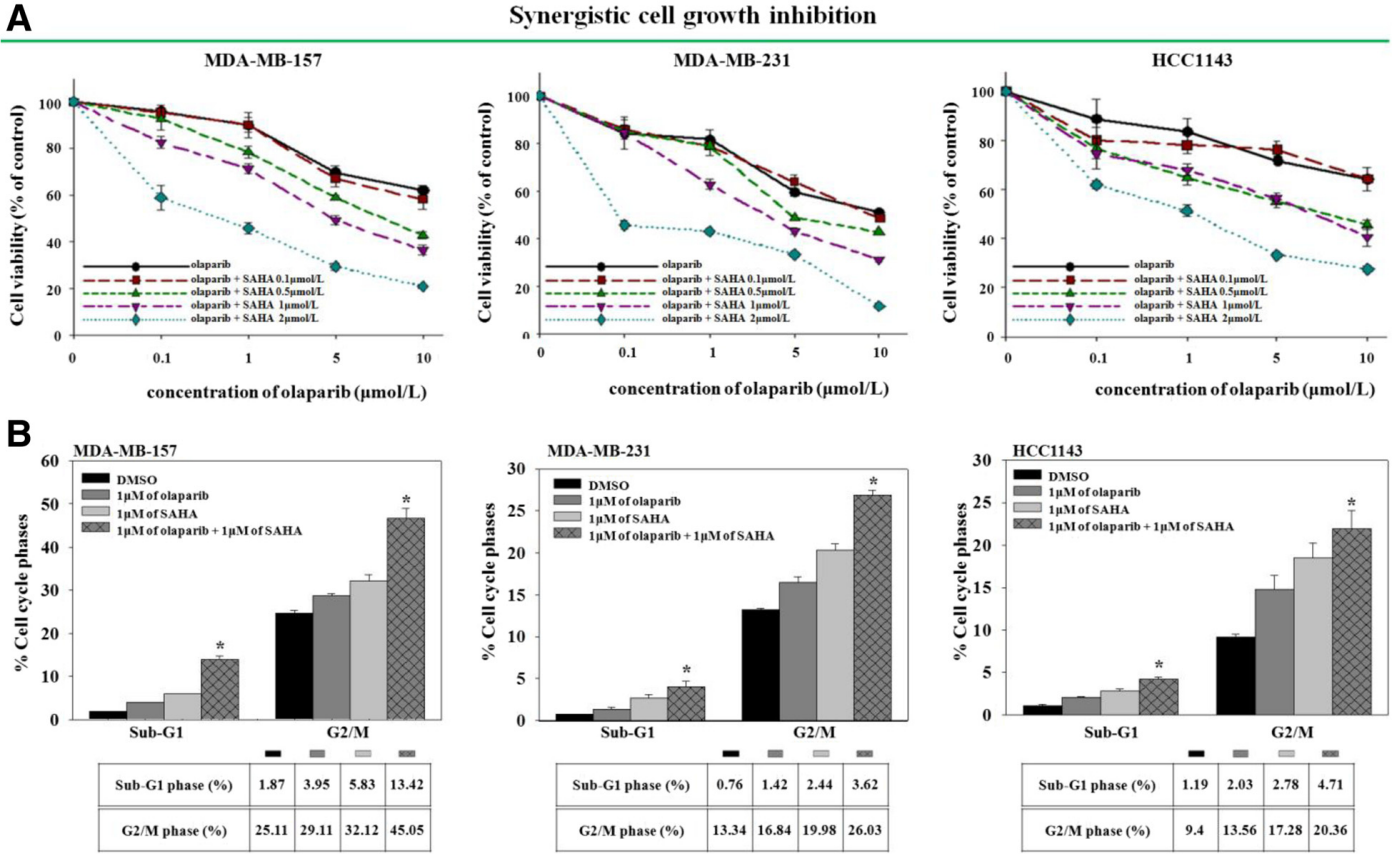
**Different sensitivity levels of triple-negative breast cancer (TNBC) cells to the co-administration of poly (ADP-ribose) polymerase (PARP) and histone deacetylase** (**HDAC) inhibitors. (A)** The cells were exposed to increasing doses of olaparib with a fixed concentration of suberoylanilide hydroxamic acid (SAHA) for 5 d. Cell survival was measured and the results are presented in a graph. **(B)** Cells were treated with olaparib and SAHA alone or in combination at the indicated concentrations for 5 d. DNA contents of the cells were analyzed with fluorescence-activated cell sorting. The proportion of cells undergoing the G2/M phase and apoptosis is presented in bar graphs. Columns represent the mean of three independent experiments and are shown with error bars (± SE); *P <0.001.

HDAC inhibition regulates the expression levels of HRR proteins, and some studies have shown that HDAC inhibition induces DNA damage [21,23]. To determine whether SAHA can enhance the growth inhibition of breast cancer cells by olaparib, various breast cancer cell lines were treated with different concentrations of SAHA or olaparib alone or in combination. IC_50_ values for each treatment and the CI index were calculated (Additional file 2: Table S1). There was a varied level of response of the combination in TNBC cell lines. For example, five TNBC cell lines were exposed to increasing doses of olaparib with a fixed concentration of SAHA. Cell growth was subsequently evaluated using an MTT assay (Figure 2A and Additional file 3: Figure S2A). The results showed that co-targeting the enzymatic activities of PARP and HDACs inhibited the proliferation of MDA-MB-157, MDA-MB-231, and HCC1143 cells (Figure 2A) but not that of HCC70 or MDA-MB-468 cells (Additional file 3: Figure S2A).

To increase our understanding of the mechanisms underlying the synergistic anti-proliferative effect of SAHA and olaparib, a cell cycle analysis was conducted. The purpose of the analysis was to determine how co-treatment with olaparib and SAHA affects cell cycle progression. The result indicated that co-administration of olaparib and SAHA promoted G2/M cell cycle arrest and apoptosis in three TNBC cells with CI values <1. However, this was not observed in the other two cells with CI values >1, which indicated an antagonistic interaction (Figure 2B and Additional file 3: Figure S2B).

### Co-administration of olaparib and SAHA decreases DSB repair capacity of the sensitive TNBC cells

**Figure 3:**
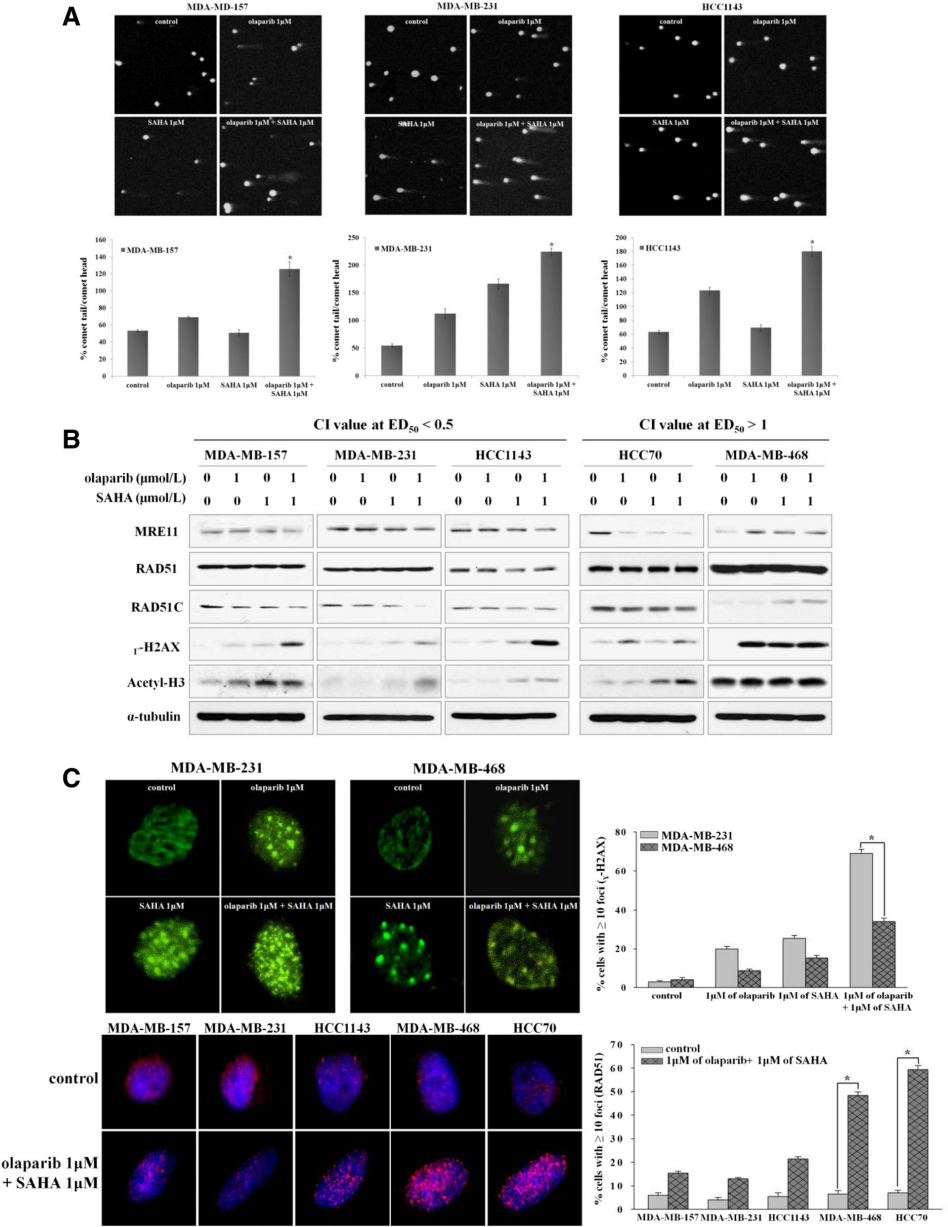
**Histone deacetylase (HDAC) inhibition enhances olaparib-induced DNA damage accumulation. (A)** Cells were treated with olaparib and suberoylanilide hydroxamic acid (SAHA) alone or in combination for 5 d. DNA double-strand breaks in the individual cells were measured with a comet assay. The percentage of tail-moment was calculated and is presented in bar graphs with error bars (± SE); *P <0.001. **(B)** The expression of DNA damage-responsive proteins was measured by western blot analysis following treatment with olaparib and SAHA alone or in combination. **(C)** The cells were treated with 1 μmol/L olaparib and/or 1 μmol/L SAHA and the immunofluorescence analysis were conducted with the indicated antibodies. Confocal microscopy was used to observe the signals corresponding to RAD51 (red) and ɣ-H2AX (green). The DNA was counterstained with 4',6-diamidino-2-phenylindole (DAPI) (blue). The percentage of cells containing more than 10 foci of RAD51 and ɣ-H2AX over three experiments is presented in a bar graph. At least 100 nuclei were analyzed for each experiment (right). Columns, the mean of three independent experiments; bars, ± SE; *P <0.001.

PARP inhibition leads to the accumulation of DNA damage [19]. HDAC inhibition also promotes DNA damage [24,25]. We hypothesized that the mechanism underlying the synergistic drug activity we observed may be due to decreased DNA double-strand break (DSB) repair capacity of TNBC cells. A comet assay was performed to assess the DNA repair ability of TNBC cells following PARP and HDAC inhibition. The results indicated that co-administration of olaparib and SAHA significantly increased the accumulation of DNA damage in sensitive TNBC cells (Figure 3A) while the DNA repair profile of the two antagonistic TNBC cells was unaffected (Additional file 4: Figure S3). Consistent with these findings, cell lines in which a synergistic effect was observed were found to have increased levels of ɣ-H2AX expression. This was seen in the sensitive cell lines with an ED_50_ value less than 0.5 following treatment with both inhibitors (Figure 3B). Furthermore, co-administration of olaparib and SAHA led to a significant reduction of RAD51 foci formation and increased the number of ɣ-H2AX foci in sensitive cell lines with a combination index at ED_50_ value less than 0.5 (Figure 3C). These data suggest a possible mechanism by which SAHA enhances cellular sensitivity to olaparib through abrogation of the DNA DSB repair pathway.

### PTEN expression influences the synergistic effects of olaparib and SAHA in TNBC cells

**Figure 4:**
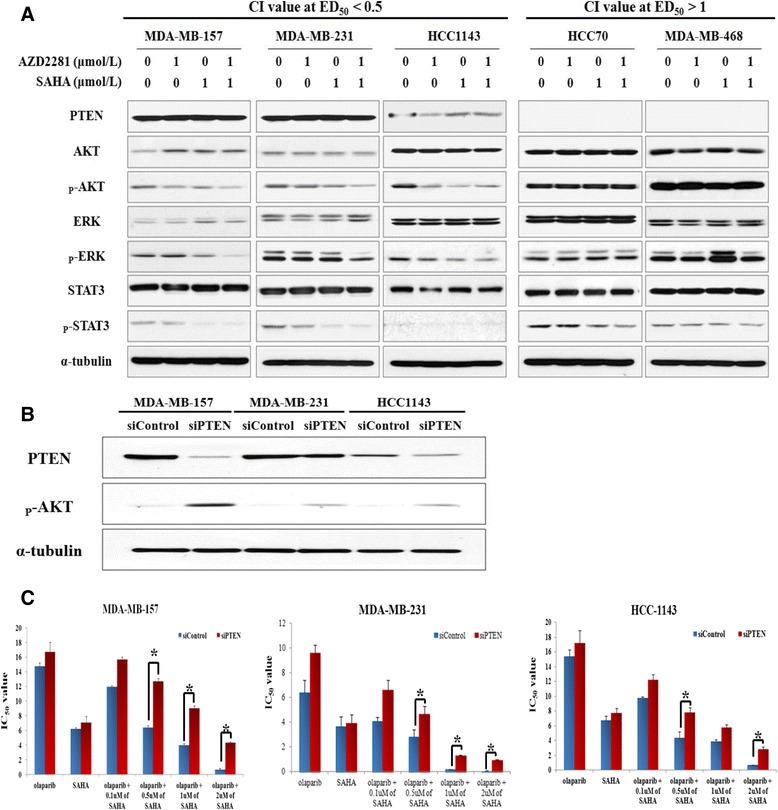
**Phosphatase and tensin homolog (PTEN) expression affects the combined effect of olaparib and suberoylanilide hydroxamic acid (SAHA) in triple-negative breast cancer (TNBC) cells. (A)** The expression levels of proliferative signaling pathway proteins in TNBC cells were analyzed by western blotting following drug treatment. **(B)** Transfection efficacy was verified by western blot analysis with anti-PTEN, anti-phosphorylated (p)-AKT, and anti-α-tubulin (as a loading control) antibodies. **(C)** PTEN silencing decreased cellular sensitivity to dual inhibition by the poly (ADP-ribose) polymerase (PARP) and histone deacetylase (HDAC) inhibitors. The cells were transfected with nonspecific control or PTEN-specific siRNA, and then exposed to increasing concentrations of olaparib with a fixed concentration of SAHA for 5 d. IC_50_ values are presented in bar graphs with error bars (n = 3); *P <0.001. CI, combination index.

To evaluate the effects of simultaneous PARP and HDAC inhibition on the proliferative signaling pathway, a western blot analysis was conducted. Interestingly, no synergy but rather, antagonism was seen in two cell lines deficient for PTEN. In addition, it is also worth noting that treatment of cells with olaparib and SAHA synergistically downregulated the levels of p-AKT and p-ERK only in the sensitive cell lines that expressed PTEN protein (Figure 4A). We therefore hypothesized that PTEN expression determines the anti-proliferative effects of the PARP and HDAC inhibitor combination in sensitive cells. To determine whether sensitivity to olaparib and SAHA co-treatment is a direct result of PTEN deficiency, we measured the IC_50_ values of olaparib with a fixed concentration of SAHA in two synergistic cell lines that were transfected with siRNA targeting PTEN or non-specific control siRNA. Successful knockdown of PTEN expression was validated by western blot analysis (Figure 4B). Data from this experiment revealed that downregulation of PTEN expression correlates with increased TNBC cell resistance to the olaparib and SAHA combination (Figure 4C).

### Autophagic cell death is induced by dual inhibition of PARP and HDAC and modulated by PTEN expression

**Figure 5:**
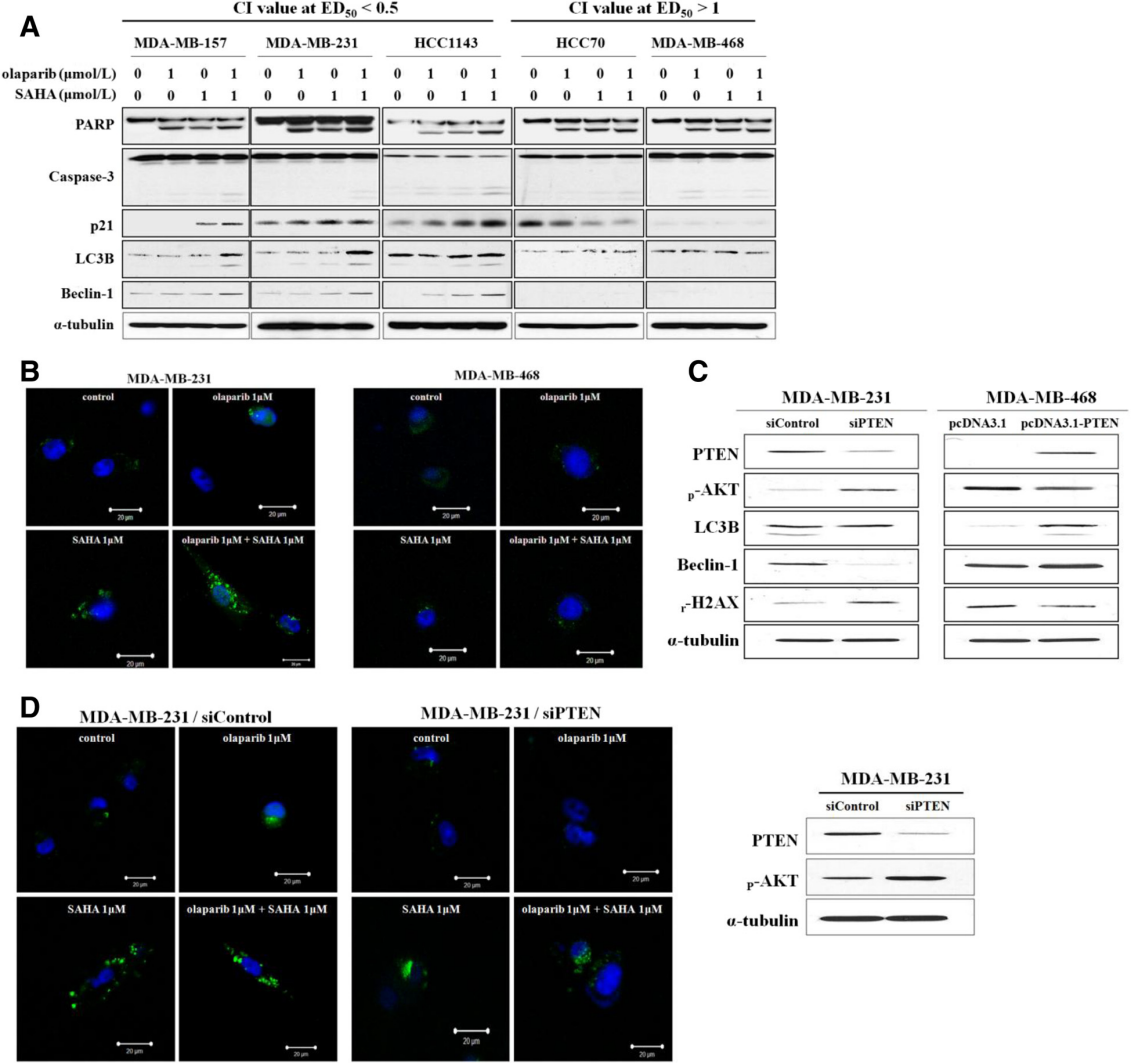
**Phosphatase and tensin homolog (PTEN) expression increases the synergistic effect of olaparib with suberoylanilide hydroxamic acid (SAHA) due to the induction of autophagic cell death. (A)** The cells were treated with olaparib and SAHA alone or in combination for 5 d. The expression levels of apoptosis and autophagy mediators were then examined by western blotting. **(B)** Induction of autophagy was confirmed by monitoring GFP-tagged LC3 expression in MDA-MB-231 (left) and MDA-MB-468 (right) cells following exposure to olaparib, SAHA, or both inhibitors. **(C)** MDA-MB-468 cells were transfected with siRNA targeting PTEN or the negative control. Additionally, the cells were transfected with an empty vector or one encoding PTEN. After 2 d the expression of autophagy markers was evaluated using immunoblotting. **(D)** Translocation of GFP-tagged LC3 in MDA-MB-231 cells transfected with control or PTEN-specific siRNA was examined by confocal microscopy (top)*.* siRNA-mediated reduction of PTEN expression was confirmed by western blotting (bottom)*.* CI, combination index.

It was unclear how PTEN expression can determine the sensitivity of TNBC cells to olaparib and SAHA. One possible explanation is that HDAC inhibition induces autophagic cell death through the regulation of PTEN expression. Therefore, SAHA-induced autophagic cell death may enhance the cytotoxic effect of PARP inhibition in cell lines that express PTEN and induce autophagic cell death. In order to test this possibility, we monitored the effects of olaparib and SAHA alone or in combination on the expression of factors associated with both apoptotic and autophagic cell death. As shown in Figure 5A, increased levels of LC3B and Beclin-1, two autophagy markers, were observed after dual inhibition of PARP and HDACs in TNBC cells that expressed PTEN. Induction of autophagy by a combination of olaparib and SAHA was further confirmed by monitoring GFP-tagged LC3 expressed in sensitive MDA-MB-231 cells and resistant MDA-MB-468 cells (Figure 5B). In the MDA-MB-468 cells, GFP-LC3B localization was cytosolic and diffuse (Figure 5B). In contrast, co-treatment with olaparib and SAHA resulted in the re-localization of GFP-LC3 into punctuate structures corresponding to autophagosomes in MDA-MB-231 cells (Figure 5B).

To determine whether increased levels of autophagy following olaparib and SAHA co-treatment is a direct result of PTEN deficiency, changes in the expressions of autophagy markers as well as autophagosome formation were assessed in MDA-MB-231 cells that were transfected with control or PTEN-specific siRNA. Downregulation of LC3B and Beclin-1 expression was clearly observed in the PTEN-knockdown MDA-MB-231 cells. Conversely, LC3B and Beclin-1 expression was induced in MDA-MB-468 cells that transiently over-expressed PTEN (Figure 5C). Consistent with the western blot data, co-administration of both inhibitors increased cytosolic autophagosome formation in MDA-MB-231 cells transfected with control siRNA but not with PTEN-specific siRNA. This appeared as a transition from diffuse cytosolic to punctuate distribution of LC3B (Figure 5D). PTEN protein expression was depleted by siRNA (Figure 5D). Taken together, these data indicate that the expression of PTEN plays an important role in determining the combined effect of olaparib and SAHA in TNBC cells. Moreover, the synergistic effect of olaparib and SAHA is associated with increased levels of both apoptosis and autophagy, two processes that are regulated by PTEN.

### Co-treatment with olaparib and SAHA significantly inhibits cell proliferation and induces both apoptosis and autophagic cell death in an *in vivo* mouse model

**Figure 6:**
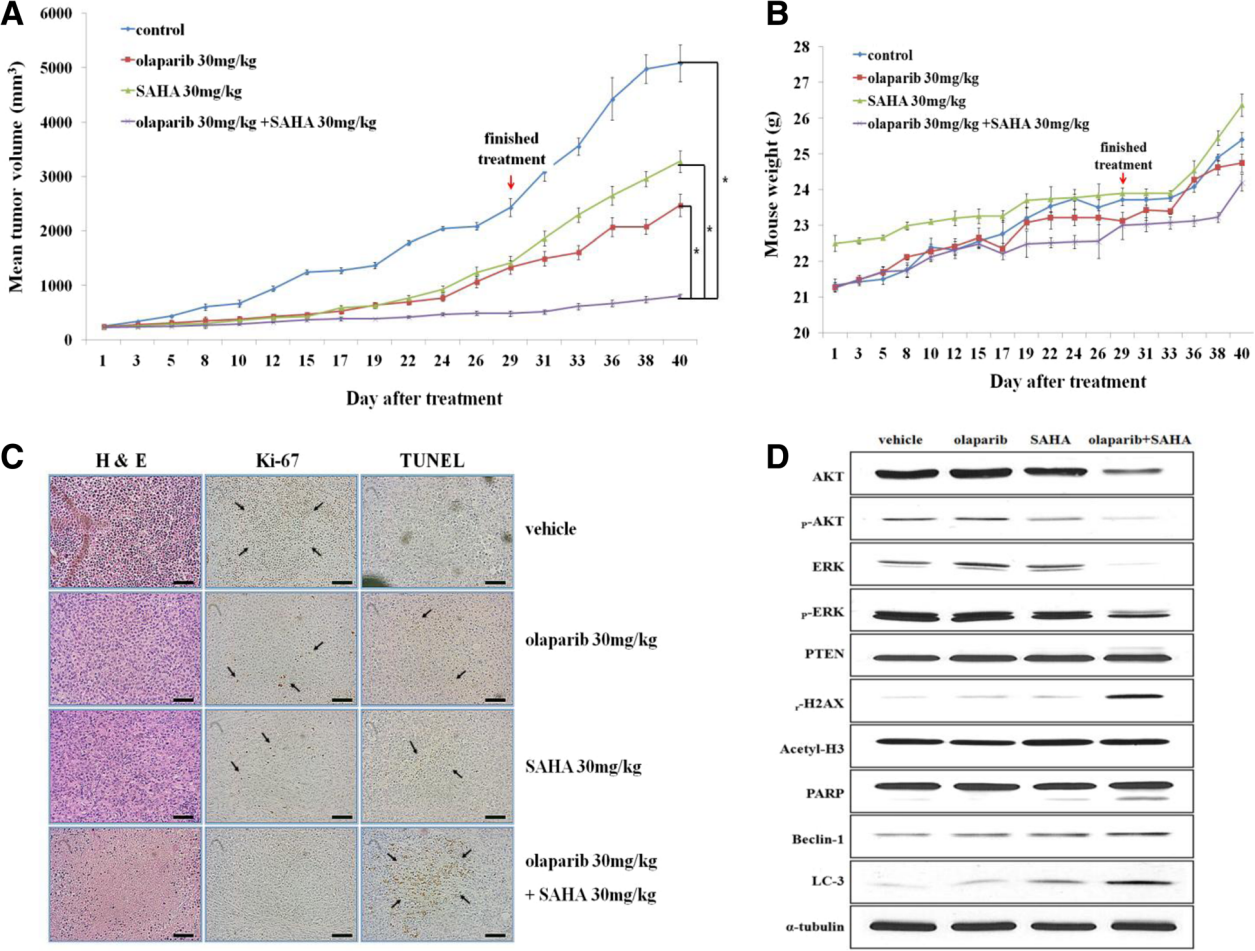
**Suberoylanilide hydroxamic acid (SAHA) enhances the anti-tumor effects of olaparib in an MDA-MB-231 xenograft model. (A)** A mouse xenograft model with MDA-MB-231 human breast cancer cells was established. The mice were treated with 30 mg/kg olaparib (n = 8), 30 mg/kg SAHA (n = 8), 30 mg/kg olaparib plus 30 mg/kg SAHA (n = 8), or vehicle alone (n = 8) daily for 28 d. Tumor volumes for each mouse were measured and are presented in a graph with the SD. Olaparib plus SAHA significantly inhibited tumor growth in a MDA-MB-231 mouse xenograft model (*P <0.001). **(B)** Changes in mouse body weight were measured to estimate the toxicity of each treatment. **(C)** The tumors were removed from the mice 10 d after drug treatment ended, and immunohistochemical staining for Ki-67 along with a TUNEL assay were conducted. Representative images from this study are presented with scale bars representing 25 μM (400× magnification). Arrows indicate positive staining. **(D)** Total cell proteins were extracted from tissues and the expression of molecules associated with proliferation, apoptosis, and autophagy were evaluated with western blotting.

To confirm our *in vitro* findings in an *in vivo* setting, we used a mouse xenograft model injected with MDA-MB-231 human breast cancer cells. Co-treatment with olaparib and SAHA significantly delayed tumor growth not only during treatment but also after treatment had ceased (Figure 6A). There were no signs of toxicity in the mice undergoing extended treatment (Figure 6B). Tumor tissues from the mice treated with both olaparib and SAHA showed lower Ki-67 expression, suggesting a reduced proliferation ability compared to the tumor tissues from mice treated with a single agent alone. This effect was associated with increased apoptosis observed with a TUNEL assay (Figure 6C). We also observed that the expression of proteins related to proliferation (such as AKT and ERK) was reduced. Additionally, the levels of PARP cleavage (associated with apoptosis) as well as LC3B and Beclin-1 (that affect the induction of autophagy) were clearly increased following co-treatment with olaparib and SAHA (Figure 6D). This experiment demonstrated that co-treatment with olaparib and SAHA significantly inhibits cell proliferation and induces both apoptosis and autophagic cell death in an *in vivo* mouse model.

## Discussion

Genomic instability is a key feature of cancer development, and DNA repair pathways have a significant impact on genomic stability. Defects in genome stability increase the sensitivity of cells to DNA damaging agents and provide an *Achilles heel* for cancer therapeutics [26,27]. Olaparib, a PARP inhibitor that targets defects in the DNA repair pathway, has produced promising results in TNBC patients with BRCA deficiencies or BRCAness. However, the population of BRCAness in TNBC patients is reported to be limited, so many efforts have been made to extend the usage of PARP inhibitors [19,28-30]. Various reports have demonstrated that compromised HRR activity sensitizes BRCA-proficient cancers to PARP inhibitors [10,19,29]. Additionally, PARP inhibitors are a useful therapeutic strategy treating cases of cancer with a variety of HRR pathway deficiencies. Recent studies have suggested that the inhibition of HDAC activity impedes the HRR pathway, resulting in increased cellular sensitivity to DNA-damaging agents [13,31]. Thus, HDAC inhibition leads to the creation of cells that may mimic an HRR-deficient phenotype, resulting in increased PARP inhibitor sensitivity [31,32].

In the present study, we evaluated the synergistic effects of simultaneous PARP and HDAC inhibition on proliferation and cell cycle progression in sensitive TNBC cell lines. We also assessed the synergistic effects of PARP and HDAC co-targeting in TNBC cells. Our findings indicated that these effects are attributable to decreased DSB repair capacity due to HDAC inhibition, thereby resulting in DNA damage accumulation induced by PARP inhibition. We also discovered that TNBC cells showed different responses to the combination of PARP plus SAHA.

Interestingly, TNBC cells exhibiting synergistic responses to the olaparib-SAHA combination had a greater decrease of proliferative pathway activity observed as AKT and ERK phosphorylation. Our findings support the hypothesis that the synergistic effects on TNBC cells depend on PTEN expression.

PTEN is a well known target of HDACs. Not surprisingly, HDAC inhibition leads to the upregulation of PTEN expression [13,14]. Even though PTEN deficiency has been suggested as a marker that can help predict positive responses to PARP inhibitors [33], the sensitivity of PARP inhibition is not associated with PTEN deficiency in at least two TNBC cell lines (HCC70 and MDA-MB-468) that we evaluated. Rather, PTEN deficiency appears to induce resistance to the combination effect of simultaneous inhibition of PARP and HDACs in TNBC cells. Activation of PTEN along with decreased AKT and ERK phosphorylation by treatment with olaparib plus SAHA in the present study suggested that proliferative signaling pathways are modulated by the combination of olaparib and SAHA. PTEN activation blocks cell cycle progression, thereby suppressing tumor formation and progression. In addition, PTEN is crucial for regulating and maintaining PI3K/AKT signaling [34]. Loss of PTEN function mainly leads to over-activation of the PI3K/AKT pathway that is frequently observed in breast cancer. The PI3K/AKT pathway represents a mechanism of resistance to cancer therapeutic agents as well as PARP inhibitors [34]. Therefore, upregulated PTEN expression induced by HDAC inhibition would enhance the cytotoxic effect of PARP inhibitors in PARP inhibitor-resistant breast cancer cells. This would be a rational argument for administering a combination regimen of olaparib plus SAHA for treating TNBC.

Another novel finding from the current investigation is that PTEN expression can determine the combined effects via the regulation of autophagic cell death. Induction of autophagy was clearly observed in TNBC cells expressing PTEN in which synergism between olaparib and SAHA was observed. Autophagy is a ubiquitous process of recycling cellular compartments and is mainly considered a cytoprotective response to metabolic stresses [35-38]. While autophagy is characterized as a mediator of cell death in the presence of chronic stress, it is unclear under which conditions autophagy promotes cell death or cell survival. Additionally, the interaction between autophagy and apoptosis is not well-established. Nevertheless, the effect of HDAC inhibition on autophagy has been studied in several types of cancers although many questions remain as to whether the induction of autophagy is cytoprotective or cytotoxic for cancer cells [38]. In general, many studies in the field of cancer therapy have focused on a cell survival mechanism of autophagy in tumor cells [37-39]. Subsequently, autophagy suppression has been suggested to be a way to improve the therapeutic benefit of cancer treatments. It has also been hypothesized that autophagy induced by HDAC inhibition enhances the ability of cancer cells to escape cell death. However, we found that increased levels of autophagy correlated with increased cell death following olaparib and SAHA combination treatment. Based on data from the present study, we suggest a mechanism by which HDAC inhibition following SAHA treatment increases PTEN expression, leading to the downregulation of proliferative signaling pathways including the AKT/mTOR cascade and an associated increased sensitivity to PARP inhibitor-induced apoptosis. In addition, HDAC inhibition contributes to autophagy induction that also results in increased cancer cell death.

In summary, findings from the current investigation demonstrated that TNBC cells have different responses to olaparib and SAHA alone or in combination. Combination therapy with selective PARP and HDAC inhibitors may be an effective strategy for treating cases of TNBC with functional PTEN expression. The combination of PARP and HDAC inhibitors significantly promoted growth inhibition as a result of proliferative signaling pathway suppression, and also led the accumulation of DNA damage. Our data suggest that the combination of PARP and HDAC inhibitors also induces both apoptotic and autophagic cell death, which increases the cytotoxic effects of the inhibitors. These combined effects resulting in cell death are regulated by PTEN expression in TNBC cells. Results from our investigation indicate that olaparib plus SAHA could be a novel strategy for treating cases of TNBC with PTEN expression. In light of these findings, the combination of PARP and HDAC inhibitors may merit further clinical evaluation in patients suffering from TNBC.

## Conclusion

Our data show that a combination of olaparib with SAHA exerted synergistic effects against TNBC cells that expressed PTEN. This combination benefit was also observed *in vivo* using an MDA-MB-231 xenograft model and the mechanism underlying the combined effects we observed was further elucidated. Our data provide a strong rationale for using a combination of olaparib with SAHA to treat TNBC patients, especially cases with functional PTEN expression.

## Additional files

Additional file 1: Figure S1. Effects of histone deacetylase (HDAC) inhibition on protein expression in triple-negative breast cancer (TNBC) cell lines. The cells were exposed to increasing doses of suberoylanilide hydroxamic acid (SAHA) for 3 d. The expression levels of DNA repair molecules were then analyzed by western blotting.
Additional file 2: Table S1. Combined effects of olaparib and SAHA on human breast cancer cell lines. The cells were treated with olaparib and suberoylanilide hydroxamic acid (SAHA) alone or in combination for 5 d. Cell viability was then calculated. IC_50_ values for each treatment and the combination index (CI) were calculated. The results are presented in the table.
Additional file 3: Figure S2. Combination of olaparib and suberoylanilide hydroxamic acid (SAHA) suppresses proliferation and cell cycle progression in insensitive triple-negative breast cancer (TNBC) cells. (**A**) An MTT cell viability assay was conducted to compare responses of the cells to increasing concentrations of olaparib with a fixed concentration of SAHA for 5 d. (**B**) The percentage of cells undergoing apoptosis and G2/M arrest following 5 d of treatment was determined by fluorescence-activated cell sorting (FACS).
Additional file 4: Figure S3. Dual inhibition of poly (ADP-ribose) polymerase (PARP) and histone deacetylases (HDACs) does not lead to the accumulation of DNA damage in insensitive triple-negative breast cancer (TNBC) cells. The levels of DNA double-strand breaks were measured with a comet assay. The percentage of tail-moment was calculated and is shown in bar graphs with error bars (± standard error).

## Abbreviations

CI: combination index
DSB: double-strand break
FACS: fluorescence-activated cell sorting
FBS: fetal bovine serum
H&E: hematoxylin and eosin
HDAC: histone deacetylase
HER2: human epidermal growth factor receptor-2
HRR: homologous recombination repair
PARP: poly (ADP-ribose) polymerase
PBS: phosphate-buffered saline
PTEN: phosphatase and tensin homolog
SAHA: suberoylanilide hydroxamic acid
TNBC: triple-negative breast cancer
TUNEL: terminal deoxynucleotidyl transferase dUTP nick end labeling
