# Supplementary figures and images for: Histone deacetylase inhibitor, suberoylanilide hydroxamic acid (SAHA), enhances anti-tumor effects of the poly (ADP-ribose) polymerase (PARP) inhibitor olaparib in triple-negative breast cancer cells
Source: Breast Cancer Res. 2015 Mar 7;17:33. doi: 10.1186/s13058-015-0534-y (PMC4425881; doi:10.1186/s13058-015-0534-y)

Figure S1

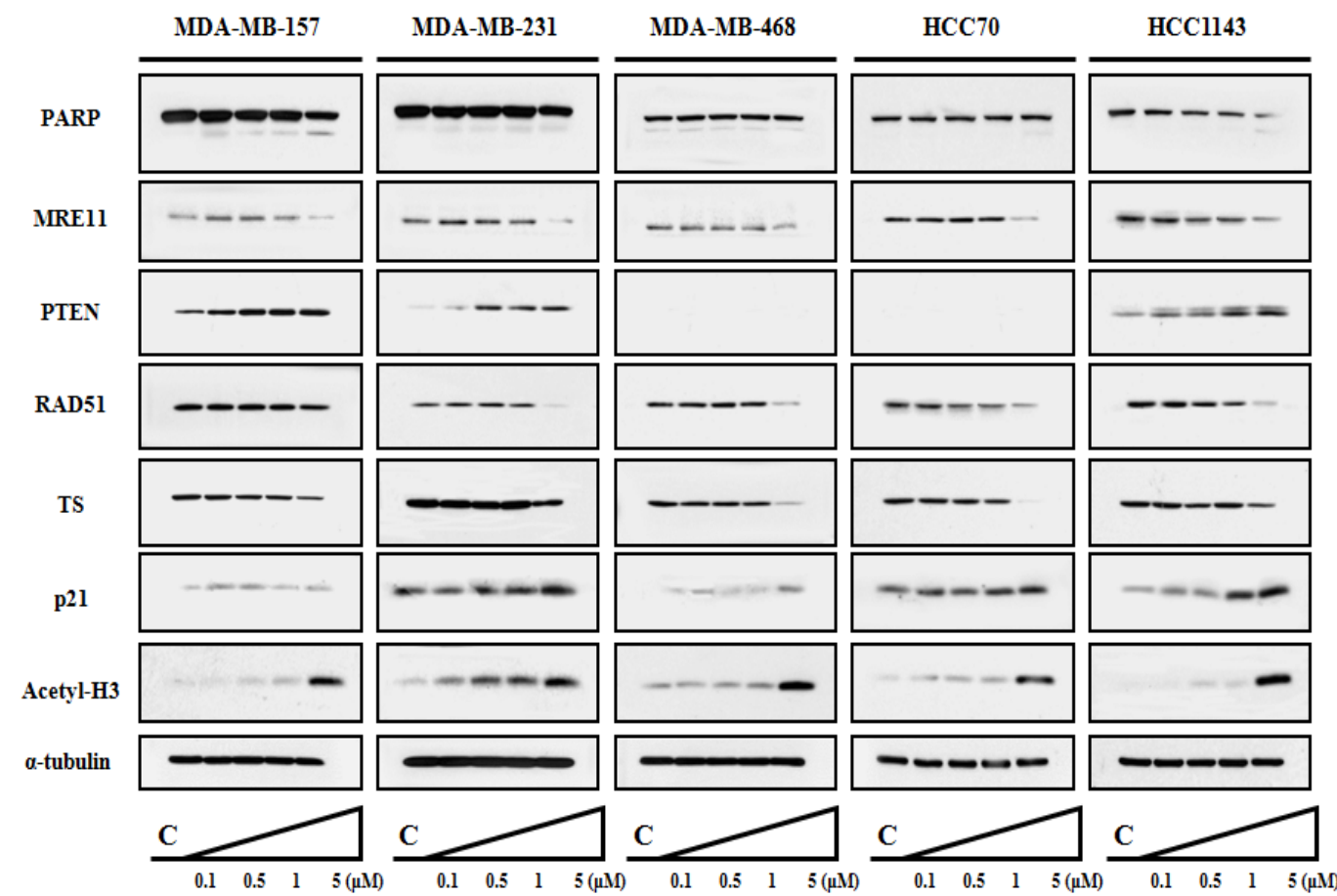

Supplement: Additional file 1: Figure S1. — Effects of histone deacetylase (HDAC) inhibition on protein expression in triple-negative breast cancer (TNBC) cell lines. The cells were exposed to increasing doses of suberoylanilide hydroxamic acid (SAHA) for 3 d. The expression levels of DNA repair molecules were then analyzed by western blotting. [file 13058_2015_534_MOESM1_ESM.pdf]

Figure S2

A.

Antagonistic cell growth inhibition

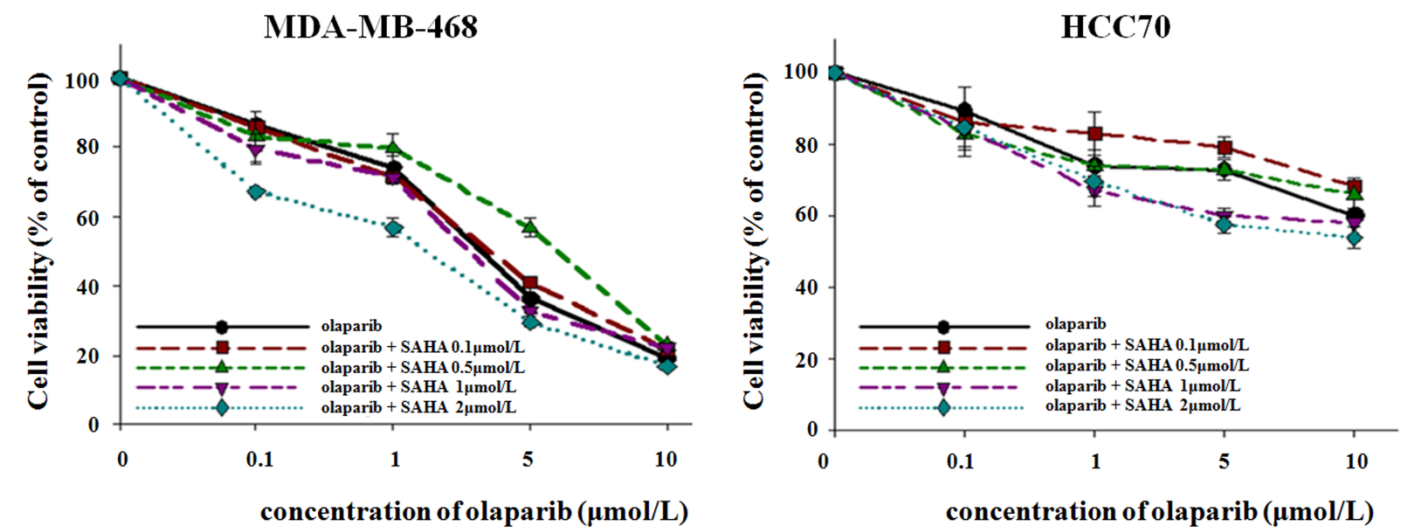

B.

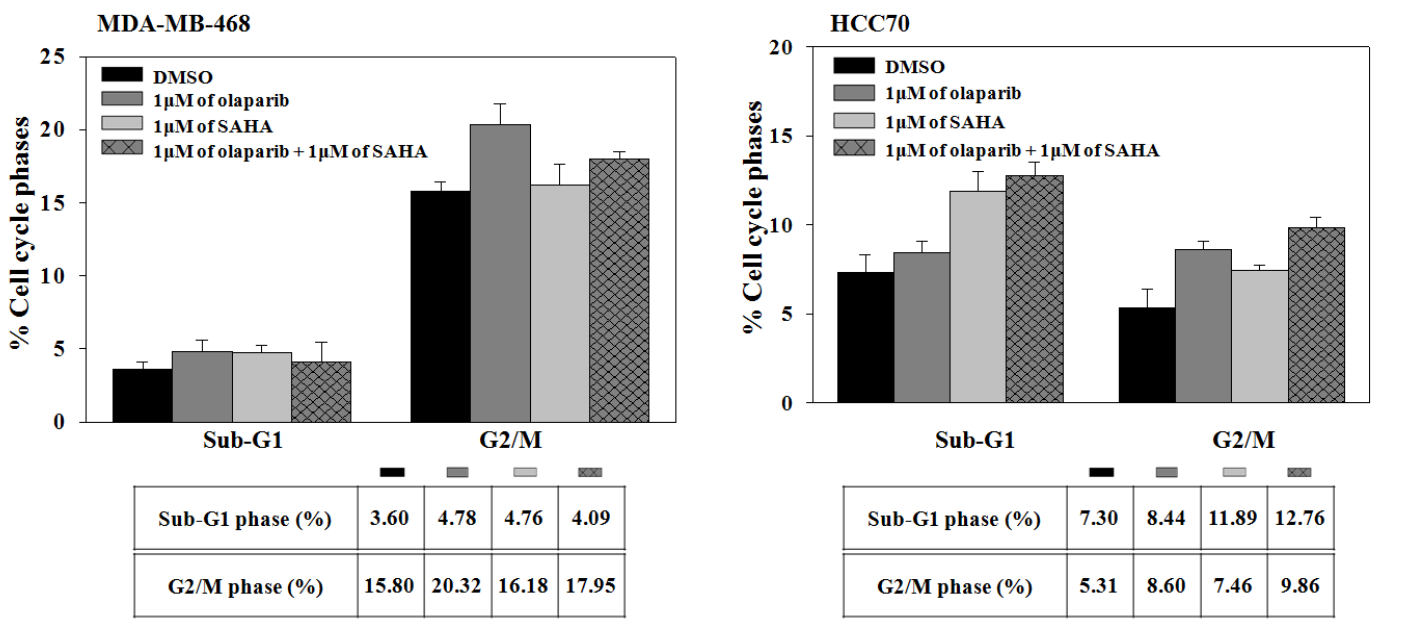

Supplement: Additional file 3: Figure S2. — Combination of olaparib and suberoylanilide hydroxamic acid (SAHA) suppresses proliferation and cell cycle progression in insensitive triple-negative breast cancer (TNBC) cells. (A) An MTT cell viability assay was conducted to compare responses of the cells to increasing concentrations of olaparib with a fixed concentration of SAHA for 5 d. (B) The percentage of cells undergoing apoptosis and G2/M arrest following 5 d of treatment was determined by fluorescence-activated cell sorting (FACS). [file 13058_2015_534_MOESM3_ESM.pdf]

Figure S3

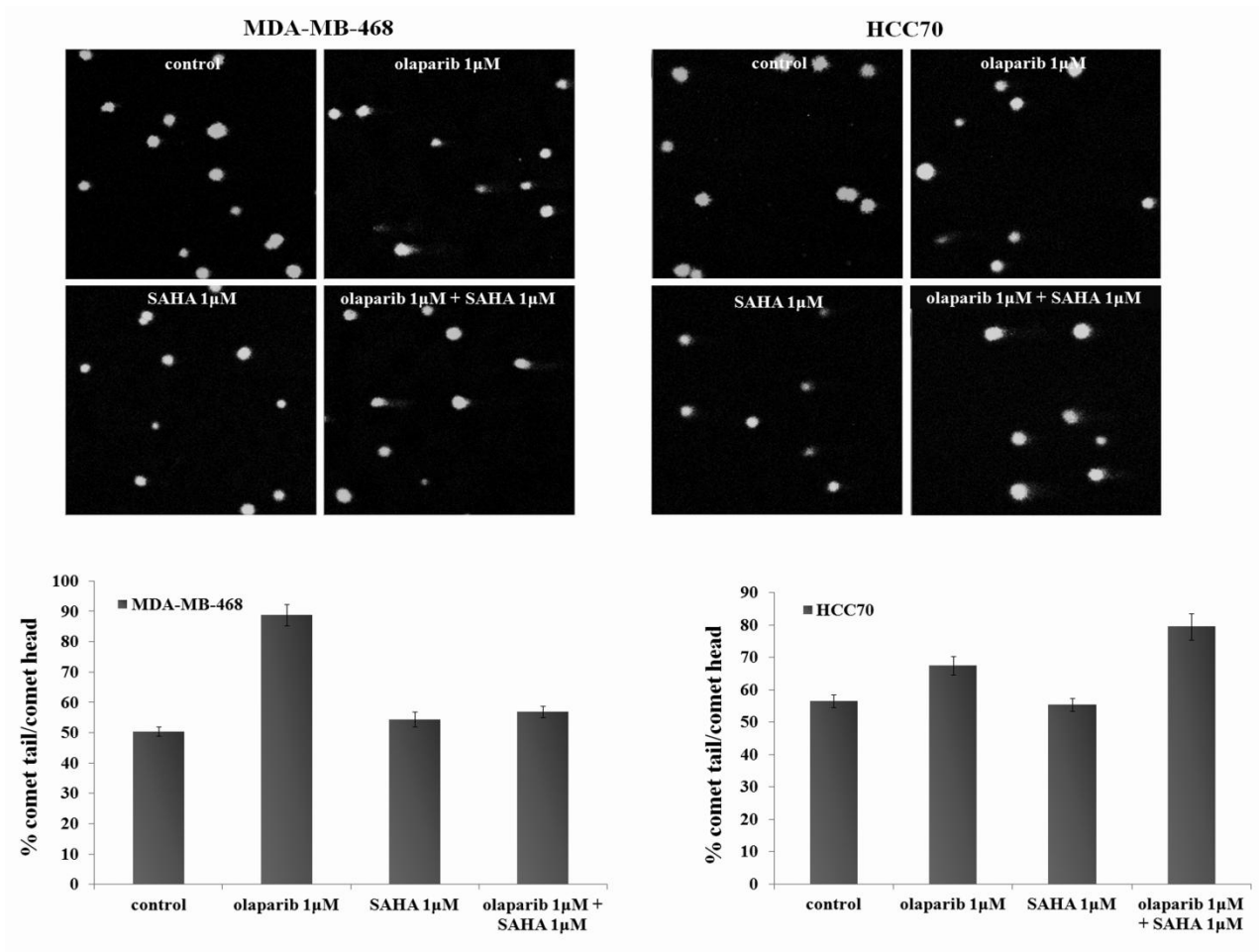

Supplement: Additional file 4: Figure S3. — Dual inhibition of poly (ADP-ribose) polymerase (PARP) and histone deacetylases (HDACs) does not lead to the accumulation of DNA damage in insensitive triple-negative breast cancer (TNBC) cells. The levels of DNA double-strand breaks were measured with a comet assay. The percentage of tail-moment was calculated and is shown in bar graphs with error bars (± standard error). [file 13058_2015_534_MOESM4_ESM.pdf]
