# Supplementary material for: Histone deacetylase inhibitor, suberoylanilide hydroxamic acid (SAHA), enhances anti-tumor effects of the poly (ADP-ribose) polymerase (PARP) inhibitor olaparib in triple-negative breast cancer cells
Source: Breast Cancer Res. 2015 Mar 7;17:33. doi: 10.1186/s13058-015-0534-y (PMC4425881; doi:10.1186/s13058-015-0534-y)
Supplement: Additional file 2: Table S1. — Combined effects of olaparib and SAHA on human breast cancer cell lines. The cells were treated with olaparib and suberoylanilide hydroxamic acid (SAHA) alone or in combination for 5 d. Cell viability was then calculated. IC50 values for each treatment and the combination index (CI) were calculated. The results are presented in the table. [file 13058_2015_534_MOESM2_ESM.pdf]

Table S1

Combined effects of olaparib and SAHA on human breast cancer cell lines							
Cell lines	BRCA1	BRCA2	PTEN	Olaparib IC_{50} (μmol/L, mean ± SD)	SAHA IC_{50} (μmol/L, mean ± SD)	1:1 combination IC_{50} (μmol/L, mean ± SD)	Combination index (ED50)
MDA-MB-157	WT	WT	WT	>10	6.9 ± 0.42	2.23 ± 0.17	0.37953
MDA-MB-231	WT	WT	WT	6.34 ± 0.17	5.31 ± 0.27	0.99 ± 0.01	0.28676
MDA-MB-468	WT	WT	Loss	3.63 ± 0.11	1.12 ± 0.07	1.59 ± 0.24	1.94014
BT-549	WT	WT	Low expression	4.72 ± 0.06	2.74 ± 0.06	0.74 ± 0.12	0.70756
HCC70	WT	WT	Loss	>10	1.78 ± 0.01	0.84 ± 0.04	1.42551
HCC1143	WT	WT	WT	>10	6.71 ± 0.14	2.02 ± 0.06	0.36249
Hs578T	WT	WT	WT	>10	3.81 ± 0.31	1.76 ± 0.08	0.9453
T47D	WT	WT	WT	>10	1.94 ± 0.02	1.31 ± 0.07	2.88855
MCF7	WT	WT	WT	>10	1.84 ± 0.08	0.66 ± 0.02	0.99978
SK-BR-3	WT	WT	WT	>10	2.87 ± 0.13	0.77 ± 0.08	0.42673
MDA-MB-453	WT	WT	WT	5.39 ± 0.54	0.97 ± 0.01	0.96 ± 0.07	2.2001
MCF10A	WT	WT	WT	>10	>10	>10	1573
